# OPALS: A New Osimertinib Adjunctive Treatment of Lung Adenocarcinoma or Glioblastoma Using Five Repurposed Drugs

**DOI:** 10.3390/cells10051148

**Published:** 2021-05-10

**Authors:** Richard E. Kast, Marc-Eric Halatsch, Rafael Rosell

**Affiliations:** 1 IIAIGC Study Center, Burlington, VT 05408, USA; 2 Department of Neurosurgery, Cantonal Hospital of Winterthur, 8400 Winterthur, Switzerland; marc-eric.halatsch@ksw.ch; 3 Dr. Rosell Oncology Institute (IOR), Quirón-Dexeus University Institute, 08028 Barcelona, Spain; rrosell@oncorosell.com

**Keywords:** EGFR, NSCLC, osimertinib, EGFR, repurposing, cancer stem cells, glioblastoma

## Abstract

Background: Pharmacological targeting aberrant activation of epidermal growth factor receptor tyrosine kinase signaling is an established approach to treating lung adenocarcinoma. Osimertinib is a tyrosine kinase approved and effective in treating lung adenocarcinomas that have one of several common activating mutations in epidermal growth factor receptor. The emergence of resistance to osimertinib after a year or two is the rule. We developed a five-drug adjuvant regimen designed to increase osimertinib’s growth inhibition and thereby delay the development of resistance. Areas of Uncertainty: Although the assembled preclinical data is strong, preclinical data and the following clinical trial results can be discrepant. The safety of OPALS drugs when used individually is excellent. We have no data from humans on their tolerability when used as an ensemble. That there is no data from the individual drugs to suspect problematic interaction does not exclude the possibility. Data Sources: All relevant PubMed.org articles on the OPALS drugs and corresponding pathophysiology of lung adenocarcinoma and glioblastoma were reviewed. Therapeutic Opinion: The five drugs of OPALS are in wide use in general medicine for non-oncology indications. OPALS uses the anti-protozoal drug pyrimethamine, the antihistamine cyproheptadine, the antibiotic azithromycin, the antihistamine loratadine, and the potassium sparing diuretic spironolactone. We show how these inexpensive and generically available drugs intersect with and inhibit lung adenocarcinoma growth drive. We also review data showing that both OPALS adjuvant drugs and osimertinib have data showing they may be active in suppressing glioblastoma growth.

## 1. Introduction

OPALS is a simple repurposed drug adjuvant regimen to osimertinib (Tagrisso^®^), aiming to retard metastatic non-small cell lung cancer’s (NSCLC) growth. It may have applicability particularly as an adjuvant to osimertinib, and in other cancers, such as glioblastoma (GB). The five OPALS adjuvant drugs are cyproheptadine, pyrimethamine, azithromycin, loratadine, and spironolactone. 

Although none of the five adjuvant drugs are currently used individually to treat cancer, they all have a preclinical sound rationale and database evidence for selective cytotoxicity to NSCLC cells. Some have preliminary clinical evidence, as well. This data is reviewed here, and it is shown how the OPALS drugs intersect with NSCLC pathophysiology to inhibit its growth. All the OPALS drugs have well-established safety records when used individually in their general medicine, non-oncology roles. General medicine physicians worldwide are familiar with all the OPALS adjuvant drugs when used in their non-oncology roles.

We reviewed core aspects NSCLC and GB growth drive and then searched the entire FDA and EMA pharmacopoeia for already-approved drugs that have data indicating they may inhibit one or another of those growth drives. Selection criteria for the OPALS augmentation regimen included (i) particularly low-risk for unpleasant side effects—this was the primary criterion, (ii) clear physiological intersection with a growth-driving element previously identified, and (iii) our clinical familiarity with the candidate drug in its non-oncology general medical role. OPALS was designed to be applicable with osimertinib as the primary treatment, with the intent to delay resistance development by increasing osimertinib’s lethality or growth suppression. Other adjuvant regimens might be more effective but at the cost of a greater risk of adverse events. 

More aggressive cancer treatment adjuvant systems using repurposed drugs, such as the ten-drug CUSP9v3 regimen for recurrent GB, may be more effective in the anti-cancer role but that comes with an attendant increase in side effect burden [1,2,3,4]. As in Palmer et al., “the 50 years old hypothesis that a curative cancer therapy can be constructed on the basis of independently effective drugs having non-overlapping mechanisms of resistance, without synergistic interaction, which has immediate significance for the design of new drug combinations” [5]; accordingly, OPALS.

OPALS is part of the repurposing movement that aims to understand the deeper pathophysiology of malignant cell growth, then look to already established drugs that might, by their primary attribute for which they are known and used or by their lesser-known ancillary attributes, intersect with the cancer’s growth mechanisms so as to inhibit them [6]. Thus, Section 3 below lists data on such intersections of the anti-allergy antihistamine cyproheptadine, the antibiotic azithromycin, the anti-protozoal pyrimethamine, the antihistamine loratadine, and the potassium-sparing diuretic spironolactone. Table 1 lists selected pharmacologic attributes of the OPALS drugs. Section 2 below is a brief introduction to osimertinib.

OPALS drugs are here hypothesized to be a generalizable adjuvant, but not alone as a primary treatment regimen, across several different cancers. OPALS was also designed particularly with osimertinib as a primary drug in mind but would be compatible with other primary treatments.

Osimertinib is FDA and EMA-approved for use in epidermal growth factor receptor (EGFR) mutated NSCLC [7]. However, as we review below, osimertinib has shown growth retardation effects also in GB and EGFR non-mutated NSCLC.

## 2. Osimertinib

Osimertinib is a third-generation, irreversible tyrosine kinase inhibitor of EGFR, EMA and FDA-approved to treat EGFR-T790M mutated NSCLC. It irreversibly binds to the EGFR kinase domain at the cysteine-797 residue in the ATP binding site, thereby blocking signaling activity [8]. A phase 3 trial (FLAURA) in EGFR T790M-mutated NSCLC compared first-line osimertinib 80 mg once daily to standard-of-care gefitinib 250 mg or erlotinib 150 mg once daily. The trial showed longer progression-free survival with osimertinib than with gefitinib or erlotinib. Median overall survival was 39 months in the osimertinib group and 32 months with gefitinib or erlotinib [8,9]. Resistance to osimertinib eventually develops, within a median of 19 months [7,10]. Importantly for the OPALS regimen, osimertinib has other kinase inhibition targets beyond EGFR, as reviewed below.

Although compared to other EGFR inhibitors, osimertinib possesses superior blood brain barrier penetration, little clinical evidence of its potential in GB is available [11,12]. An enduring puzzle in clinical GB research has been the robust data showing that EGFR, mutated or just EGFR overexpressed, commonly drives growth in both NSCLC and GB, yet older, non-osimertinib EGFR inhibitors such as erlotinib and gefitinib anti-EGFR treatments commonly benefit in NSCLC, but have utterly failed to benefit in GB [13]. An EGFR mutation, EGFRvIII is commonly found in GB and osimertinib inhibits this with an IC50 <100 nM, and in a preclinical study, osimertinib inhibited EGFRvIII-positive GB growth In Vitro and in an orthotopic xenograft model [14]. Thus, an osimertinib clinical trial in GB—with or without OPALS adjuvant—would be eminently worthwhile.

**Figure 1:**
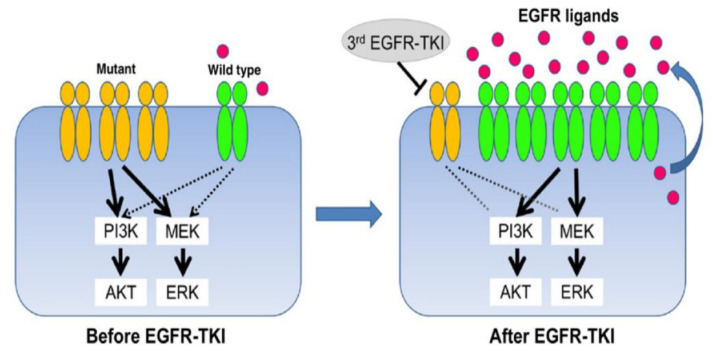
Schematic showing escape from osimertinib growth inhibition by increasing expression of non-mutated EGFR. References in text.

In principle, when osimertinib is used to treat EGFR amplified or EGFR mutated NSCLC, such resistance evolves via:

- Multiple further EGFR mutations in EGFR T790M-positive NSCLC during osimertinib that increase the IC50 of osimertinib [15];
- NSCLC that survives initial EGFR inhibitor treatment with either gefitinib or osimertinib, commonly do so through development of EGFR-independent activation of signal transducer and activator of transcription 3 (STAT3) and Src- YES-associated protein 1 (YAP1) signaling [16,17];
- Transformation to squamous or small-cell lung cancer that is not dependent on EGFR for growth [18];
- Evolution of parallel, growth-driving RTKs, including AXL, EGFR family members, and insulin growth factor 1 receptor, MET amplification, BRAF fusions, ALK fusions, Kras mutations and RET fusions [18,19,20];
- Amplification of EGFR wild-type alleles conferring resistance to osimertinib [21], schematically depicted in Figure 1.

As an example of potential circumvention of osimertinib resistance, co-targeting EGFR-T790M with osimertinib plus STAT3/Src with a non-marketed, nonpeptidic small molecule, STAT3 inhibitor aminocarbonyl-amino-5-4-fluorophenyl-3-thiophenecarboxamide, was synergistic in two EGFR-mutant NSCLC cell lines—PC9 harboring EGFR exon 19 deletion, E746-A750, and H1975 harboring both sensitizing L858R and resistant T790M mutations [16].

Clinical trials with osimertinib or other EGFR tyrosine kinase inhibitors plus Src/YAP1 inhibitors or repurposing drug regimens like OPALS are needed with the aim of overcoming or circumventing adaptive resistance mechanisms. We believe that co-treatment with OPALS or other adjuvants could circumvent or delay some of the wide array of osimertinib resistance development paths.

In addition to good brain tissue levels and inhibition of several mutated EGFRs, further benefits of osimertinib are: (A) it may be noted that osimertinib, at least partially, reverses ABCB1 and ABCG2 export of intracellular chemotherapeutic drugs like doxorubicin or temozolomide [22,23]. (B) As an example of osimertinib’s non-EGFR targets, osimertinib inhibited EGFR-negative GB cells by blocking MAPK-interacting kinases and preventing eukaryotic translation initiation factor 4E (eIF4E) phosphorylation [24].

## 3. The OPALS Medicines

### 3.1. Cyproheptadine

Although cyproheptadine is known as a potent antihistamine drug active at H1 receptor, in continuous use since the 1940s, it has multiple other inhibitory receptor bindings. These are listed in Table 2 [25]. With high inhibitory activity at serotonergic receptors 5HT2a, 5HT2b, and 5HT2c, cyproheptadine is also commonly called an anti-serotonergic drug. Cyproheptadine, 24 mg daily, normalized Cushing patient pulsatile cortisol in those without a pituitary adenoma [26]. Although there are more effective medicines in this role, cyproheptadine does increase appetite [27].

Cyproheptadine inhibited hepatocellular carcinoma (HCC) cell growth In Vitro with an IC50 of 44 microM [28]. Remissions of HCC with cyproheptadine have been reported by different groups [29,30]. A retrospective review of HCC patients of all stages showed decreased mortality in those who received palliative cyproheptadine [31]. A similar review of bladder cancer showed similar reduced mortality in those using cyproheptadine [32]. Sorafenib plus cyproheptadine-treated advanced HCC patients had a median survival of 11 months compared to 5 months in a matched HCC group on sorafenib alone [33]. 

Cyproheptadine inhibits In Vitro and xenograft growth of mantle lymphoma cells [34]. In mouse models of myeloma, cyproheptadine demonstrated inhibitory activity via cyclin D inhibition, inducing G0 arrest with subsequent apoptosis in the myeloma cells [35].

Regression of carcinoid tumors was seen in two of seven patients given cyproheptadine [36]. Others found symptomatic carcinoid improvements but no carcinoid regressions from cyproheptadine at maximum tolerable doses that ranged from 12 to 48 mg daily [37,38]. Of particular note, recent In Vitro studies demonstrated good cytotoxicity of cyproheptadine to GB cells with an IC50 of 95 microM [39].

NSCLC expresses the most proteins related to choline uptake, synthesis, transport, and degradation of acetylcholine [40]. Both nicotinic and muscarinic acetylcholine receptors are present on NSCLC and both have a large database showing agonism at these receptors form part of NSCLC’s growth drive [41,42,43].

Cyproheptadine inhibited urothelial carcinoma cells’ growth In Vitro and in a xenograft model [44]. Cell cycle arrest followed c-Myc, induction of p21 and p27, and the stabilization of Retinoblastoma protein expression [44]. Cyproheptadine decreased expression of anti-apoptotic proteins Bcl-2, Mcl-1, and XIAP and suppressed AKT activation in myeloma cells by limiting its export from nucleus [45].

Histamine signaling at the H1 receptor is a worthwhile target to inhibit in both GB and NSCLC. Histamine signaling at H1 has been a documented mitogen in GB for over 50 years [46,47,48,49,50,51].

Muscarinic acetylcholine receptors were particularly upregulated in human GB where the tumor is invading the surrounding brain. Furthermore, elevated expression of muscarinic receptors on GB biopsy material was associated with shorter patient survival [52]. A similar study in NSCLC found a similar association only for nicotinic acetylcholine receptors [53]. Therefore, cyproheptadine may be an ideal and simple adjuvant to standard GB or NSCLC treatments.

H1 signaling enhances proliferation of NSCLC cells [54]. An oddity of H1 agonism is that this usually results in upregulation of muscarinic receptors [55,56,57]. Therefore, since cyproheptadine is particularly strong at inhibition of both H1 and muscarinic receptors, it might be the ideal augmentation drug in NSCLC.

### 3.2. Azithromycin

The currently marketed macrolide antibiotics, erythromycin, clarithromycin, and azithromycin, exert their antibacterial effects by reversibly binding to the 50 s subunit of the bacterial ribosome. Basic pharmacokinetics of azithromycin are listed in Table 3.

Chu et al. studied advanced NSCLC (both adeno and squamous) given paclitaxel and cisplatin with and without low dose azithromycin (500 mg/day on days 1 to 5 of 28 day cycle). Those with azithromycin had marginal but unequivocal benefit in progression free and overall survival [58].

Mucosa-associated lymphoma patients were given oral azithromycin 1500 mg once weekly, four times a month as sole treatment. Of 16 patients treated, two had a complete remission and two experienced partial remissions [59]. Resistance to the cyclin-dependent kinase (CDK4 and CDK6) inhibitor palbociclib derives from its sequestration in lysosomes in triple negative breast cancer cells [60]. Reasoning that azithromycin concentrates in lysosomes, increasing their pH, they treated CDK4/6 inhibitor resistant TNBC cells with azithromycin that did indeed convert them to palbociclib sensitive [60].

In colon adenocarcinoma cells, azithromycin potentiated In Vitro induction of apoptosis by tumor necrosis factor-related apoptosis-inducing ligand (TRAIL) via autophagy inhibition [61]. Autophagy inhibition was also the postulated mode of action (MOA) for azithromycin cytotoxicity in squamous carcinoma cells, but this occurred only under nutrient-starved culture conditions [62]. Simultaneously targeting two major related intracellular protein degradation systems, such as the ubiquitin-proteasome with bortezomib together with autophagy-lysosome inhibition with azithromycin, enhances apoptosis in multiple myeloma cells that were resistant to either alone [63]. Temozolomide exposure induces a protective autophagy in glioma cells [64].

Azithromycin inhibited A549 lung tumor growth and tumor-related angiogenesis in a murine xenograft NSCLC model [65]. Azithromycin specifically reduced matrix metalloproteinase-9 (MMP-9) mRNA and protein levels in LPS exposed monocytic THP-1 cells. [66]. MMP-9 is part of a suite of tissue degrading enzymes active in facilitating malignant cell invasion of surrounding tissue. Increased serum MMP-9 activity correlated with advanced NSCLC, shorter survival and presence of distant metastasis [67]. MMP-9 is similarly active in promoting GB growth and invasion [68,69].

After a single preoperative oral dose of 500 mg azithromycin in humans, lung tissue contained 3.10 micrograms/g at 24 h, 2.55 micrograms/g at 72 h, and 3.13 micrograms/g at 120 h, as shown in Table 3 [70].

### 3.3. Pyrimethamine

Pyrimethamine is a 248 Da lipophilic drug used to treat malaria for over 50 years and continues in this role today in 2021. It inhibits human dihydrofolate reductase (DHFR) [71,72]. Pyrimethamine’s Ki = 38 nM at DHFR is comparable to that of methotrexate, Ki = 2.3 nM, folinic acid Ki = 320 nM, and folic acid Ki = 830 nM [71,72]. As with folate, to be active, methotrexate (MTX) must be retained within the cell; to be retained in cells, both MTX and folate must become polyglutamated. 

DHFR catalyzes NADPH-dependent reduction of 7,8-dihydrofolate to 5,6,7,8-tetrahydrofolate. MTX is a high affinity inhibitor of DHFR, commonly used in treating several cancers, that blocks DNA synthesis by disrupting the metabolism of methionine and the synthesis of S-adenosyl-methionine, purines, and thymidylate. Thymidine synthetic pathway depends on the methylation of deoxyuridine, the methyl donor being 5, lO-methylenetetrahydrofolate [72,73].

Since half of pyrimethamine-treated people will develop readily reversible bone marrow suppression, blood monitoring is required [73,74]. Of great interest in treating GB or brain metastases from breast or lung cancer is the unusual property of pyrimethamine to be concentrated in the brain at several times greater than plasma levels [75].

Reduced cellular accumulation is one of the determinants of resistance to both lipophilic antifolates, such as pyrimethamine, and hydrophilic antifolates, such as MTX. Cancer cell resistance to lipophilic antifolates seems to develop more readily than MTX due to differences in mechanism of intracellular retention. MTX is retained intracellularly by polyglutamination. Pyrimethamine is retained by its lipophilicity, but also readily exported by P-gp [72,76].

Moreover, resistance to lipophilic antifolates occurs by an increase in folate accumulation with resultant expansion of the intracellular folylpolyglutamate pool, in turn resulting in increased functioning folate competition with the nonfunctional lipophilic antifolate [76].

Table 4 lists characteristic changes seen in mammalian cells that are associated with reduced cytotoxicity of pyrimethamine. Note the dramatically increased glucose requirement associated with becoming pyrimethamine-resistant [76]. Clinically, this may work in our favor, even in the absence of direct pyrimethamine cytotoxicity.

The risks of side effects with pyrimethamine are difficult to evaluate because pyrimethamine is rarely given alone when used as an antibiotic in treating malaria or Toxoplasmosis. Bone marrow suppression, rash, and diarrhea are not uncommon (10% to 20%). These are readily reversible with dose reduction and/or folinic acid rescue [74].

In an acute myelogenous leukemia model, pyrimethamine was more effective in inhibiting growth than was MTX. In Vitro proliferation was reduced 2.5-fold at 0.1 µM and 12.7-fold at 0.5 µM [77]. Several patients with polycythaemia rubra vera and with essential thrombocythemia were successfully controlled with pyrimethamine, reported in 1987 [78].

It is unclear why early reports in the 1970s of successful pyrimethamine treatment (2 mg/kg/day for 7 days) of meningeal recurrence of acute lymphoblastic leukemia in children have not been followed up, or why such use is currently rare to non-existent [79]. Pyrimethamine in combination with thioguanine, vincristine, and dexamethasone was only partially effective in adult acute lymphocytic leukemia [80]. Adding pyrimethamine to daunorubicin, cytosine arabinoside and thioguanine failed to prevent meningeal involvement in adult acute nonlymphocytic leukemia [81].

### 3.4. Loratadine

Loratadine is an old antihistamine, of unsurpassed safety, sold over-the-counter (i.e., without need for prescription) in many jurisdictions around the world. Perhaps most importantly for loratadine use in melanoma, empirically, epidemiological study revealed better survival in melanoma patients who co-incidentally were heavy users of loratadine in the anti-allergy role [82]. This loratadine effect may not be cancer type-specific, in that a similar benefit was seen in an epidemiological study of loratadine users with breast or ovarian cancer [82,83,84].

Loratadine is a cationic amphiphilic drug, where amphiphilic refers to drugs with hydrophobic parts and hydrophilic sites within the same molecule [85,86]. As such, cationic amphiphilic drugs such as loratadine tend to accumulate at the luminal lysosome membrane, with consequent inhibition of acid sphingomyelinase and other lysosomal lipases [87]. The resultant leak of lysosomal contents into cytosol leads to cell death if the leak is severe enough [85,86]. Such a loratadine-mediated lysosomal leak as a cause of cell death was demonstrated in chronic lymphocytic leukemia [88].

Loratadine sensitized bladder and oral squamous cell carcinoma cells to microtubule disrupting drugs [89,90]. Of note, it is the invasive subset of GB cells that are particularly vulnerable to lysosomal membrane destabilization [91].

### 3.5. Spironolactone

Spironolactone is a cheap potassium-sparing diuretic used worldwide to treat hypertension and heart failure. It entered clinical practice in the 1950s. The primary action in its most common use is to block activation of the mineralocorticoid receptor (MR). Spironolactone causes a natriuretic diuresis by preventing aldosterone stimulation of the MR [92]. Spironolactone is also used in general medicine to treat acne, hair loss, and polycystic ovarian syndrome and hirsutism in women [93]. Spironolactone crosses the intact blood brain barrier at about 50% of plasma levels [94,95].

Canrenone is an active metabolite of spironolactone with full MR antagonism and a half-life several times longer than spironolactone (see Table 1) [96]. Aldosterone agonism at the MR has both genomic and non-genomic effects [97].

Nongenomic MR signaling transactivates several receptor tyrosine kinases, including EGFR, platelet derived growth factor (PDGFR), and insulin-like-growth factor 1 receptor (IGF1R) [98,99,100,101,102,103]. In a series of papers between 2007 and 2012, Grossman et al. showed that aldosterone signaling at the MR increases EGFR expression and can transactivate it, and that this is blocked by spironolactone [98,99,100,101,102,103]. Importantly, EGFR and MR co-localize on the outer cell membrane of some cells.

MR agonism by aldosterone can result in phosphorylation of the intracellular portion of unliganded EGFR in the same manner as if EGFR had dimerized and bound its cognate ligand [102,104]. In a potentially destructive reciprocal interaction, EGFR activation by ligand increases MR transcription and translation [105]—a mutually reinforcing feedback loop between the EGFR and MR. The MR can be stimulated also by glucocorticoids, and this also is blocked by spironolactone. Aldosterone activates unliganded EGFR by both MR dependent, spironolactone inhibitable, and non-MR spironolactone insensitive pathways [102,106].

An interesting intersection with renal failure, glomerular mesangial proliferation is mediated in part by aldosterone activated MR transactivating EGFR [107,108,109].

Importantly for OPALS, in 2019, experimental work again indicating the association between EGFR and the MR showed that spironolactone decreases GB cell survival and sensitized glioma cells to the EGFR inhibitor osimertinib [110]. 

Spironolactone was also shown to inhibit homologous repair of ds-DNA breaks [110,111,112,113]. The MOA of this is thought to be by spironolactone’s induction of xeroderma pigmentosum group B (XPB) protein degradation. XPB forms part of the multimeric transcription factor II-H related DNA repair process.

High throughput screening non-obvious drug combinations identified spironolactone as a synergistic partner drug to cisplatin and the related inhibition of homology directed DNA repair [114,115]. Although some studies have shown spironolactone exerts its anticancer effects by inhibiting DNA repair, thereby augmenting DNA-damaging drugs, a recent report demonstrated spironolactone’s synergy with non-genotoxic EGFR inhibitor osimertinib as well as to gemcitabine in both GB and in NSCLC cells [110].

These findings, referenced above, are representative—not all inclusive—of the extensive database showing a reciprocal relationship between the MR and the EGFR, leading to the conclusion that when the action of one is desirable to block, the action of both should be blocked, a conclusion others drew in 2011 (but we have yet to act on this) [116]. Hence, if osimertinib, then spironolactone.

## 4. Discussion

This report was not a comprehensive review of the anti-cancer growth aspects that have been demonstrated for the five OPALS drugs. The references and reviewed data were just enough to show the value and pre-safety of the regimen. The works we cite here were fairly representative of recent data on these drugs’ intersections with growth-driving elements of NSCLC and, to lesser extent, of GB.

## 5. Conclusions

Given the preclinical and clinical data reviewed here, in face of the usually fatal outcome of GB and of metastatic NSCLC and the eminently benign expected side effects from adjuvant OPALS, a pilot study of OPALS along with current standard treatment is warranted. 

## Figures and Tables

**Table 1:** Basic pharmacological attributes of the OPALS drugs, cyproheptadine, pyrimethamine, azithromycin, and spironolactone. T1/2 is the elimination half-life. References in text. All the OPALS drugs have empirical evidence of NSCLC and GB growth inhibition from preclinical studies.

Drug	T 1/2	Plasma Level	OPALS Function
Cyproheptadine	16 h	33 microg/L av 669 microg/L max	anticholinergic, Bcl-2 inhibition, antihistamine,
Pyrimethamine	4 d	500 microg/L	DHFR inhibition,
Azithromycin	2–3 d	31 mg/L	MMP-9 reduction, autophagy inhibition
Loratadine	8 h	30 μg/L	lysosomal leakage
Spironolactone canrenone	2 h 17 h	140 microg/L	EGFR transactivation DNA repair inhibition

**Table 2:** Inhibitory binding of cyproheptadine at histaminic (H), muscarinic (M), serotonergic (5HT), and dopamine (D) receptors.

Receptor	Ki nM
H1	0.06
M1	12
M2	7
M3	12
M4	8
M5	12
5HT1a	59
5HT2a	1.7
5HT2b	1.5
5HT2c	2.2
D3	8

**Table 3:** Azithromycin level after a single 500 mg oral dose. References in text.

Post-Dose	Brain Microg/g	CSF Microg/mL	Serum Microg/mL
24 h	2.63 +/− 2.58	<0.015	0.031 +/− 0.044
48 h	3.64 +/− 3.81	<0.015	0.016 +/− 0.011
72 h	0.74 +/− 0.37	<0.015	0.012 +/− 0.005
96 h	0.41	<0.015	0.008

**Table 4:** Characteristic changes seen in mammalian cells that are associated with reduced cytotoxicity of pyrimethamine.

Changes in Pyrimethamine Resistant Cancer Cells Compared to Sensitive Counterpart:
lower external folate requirement for growth
3 x increased intracellular polyglutamated folate content
increased lysosome number
increased folylpolyglutamate synthetase
increased P-gp export activity
DHFR gene amplification

## Data Availability

All data has been included in the manuscript.

## Abbreviations

CSC	cancer stem cells
DHFR	dihydrofolate reductase
GB	glioblastoma
EGFR	epidermal growth factor receptor
eIF4E	eukaryotic translation initiation factor 4E
HCC	hepatocellular carcinoma
MMP-9	matrix metalloproteinase-9
MOA	mode of action
MR	mineralocorticoid receptor
MTX	methotrexate
NSCLC	non-small cell carcinoma
